# Water Extracts from Industrial Hemp Waste Inhibit the Adhesion and Development of *Candida* Biofilm and Showed Antioxidant Activity on HT-29 Colon Cancer Cells

**DOI:** 10.3390/ijms25073979

**Published:** 2024-04-03

**Authors:** Leonardo Donati, Debora Casagrande Pierantoni, Angela Conti, Eleonora Calzoni, Laura Corte, Claudio Santi, Ornelio Rosati, Gianluigi Cardinali, Carla Emiliani

**Affiliations:** 1 Department of Pharmaceutical Sciences, University of Perugia, 06121 Perugia, Italy; leonardo.donati1@studenti.unipg.it (L.D.); debora.casagrandepierantoni@unipg.it (D.C.P.); angela.conti@unipg.it (A.C.); claudio.santi@unipg.it (C.S.); ornelio.rosati@unipg.it (O.R.); gianluigi.cardinali@unipg.it (G.C.); 2 Department of Chemistry, Biology and Biotechnology, University of Perugia, 06121 Perugia, Italy; eleonora.calzoni@unipg.it (E.C.); carla.emiliani@unipg.it (C.E.); 3 CEMIN Excellence Research Centre, 06123 Perugia, Italy

**Keywords:** Industrial *Cannabis*, Light *Cannabis*, natural products, water extracts, anti-biofilm, antioxidant, *Candida albicans*, *Candida parapsilosis*, *Candida tropicalis*

## Abstract

The evolution of regulatory perspectives regarding the health and nutritional properties of industrial hemp-based products (*Cannabis sativa* L.) has pushed research to focus on the development of new methods for both the extraction and formulation of the bioactive compounds present in hemp extracts. While the psychoactive and medicinal properties of hemp-derived cannabinoid extracts are well known, much less has been investigated on the functional and antimicrobial properties of hemp extracts. Within the hemp value chain, various agricultural wastes and by-products are generated. These materials can be valorised through eco-innovations, ultimately promoting sustainable economic development. In this study, we explored the use of waste from industrial light cannabis production for the extraction of bioactive compounds without the addition of chemicals. The five extracts obtained were tested for their antimicrobial activity on both planktonic and sessile cells of pathogenic strains of the *Candida albicans*, *Candida parapsilosis*, and *Candida tropicalis* species and for their antioxidant activity on HT-29 colon cancer cells under oxidative stress. Our results demonstrated that these extracts display interesting properties both as antioxidants and in hindering the development of fungal biofilm, paving the way for further investigations into the sustainable valorisation of hemp waste for different biomedical applications.

## 1. Introduction

*Cannabis sativa* L. (Linnaeus) originated in Asia, probably in China, and has gradually spread worldwide across various regions [1]. Over time, this plant has given rise to distinct variants, each characterized by specific phenotypic traits influenced by environmental conditions. Throughout history, Cannabis has been renowned not only for its psychoactive effects but also for its medicinal applications. Additionally, certain secondary metabolites within *Cannabis sativa* L. contribute to its antimicrobial properties, making it valuable in treating bacterial and fungal infections [2,3].

In *Cannabis*, cannabinoids take centre stage as the main secondary metabolites, showing a broad spectrum of activity and properties [4]. They predominantly accumulate within the trichomes of female inflorescences, but they are also present in other parts of the plant, including seeds and leaves [5]. Alongside cannabinoids, other compounds with pharmacological potential include flavonoids, terpenes, and alkaloids [6].

Over the past two decades, *Cannabis* has experienced a resurgence due to evolving policies and recent legislation supporting its medical use and cultivation [7,8]. Nowadays, domesticated varieties are cultivated globally, primarily for industrial purposes. The change in legislation has given new impetus to the business, driven by the recognized industrial and therapeutic potential of this plant. Many studies highlighted the various beneficial effects associated with the bioactive compounds extracted from this plant. These compounds include antioxidants [9,10,11], neuroprotective [6], antimicrobials [1], antifungals, anti-biofilm molecules [12], and nutraceuticals [13]. The terms “hemp” or “industrial hemp” are generally used to refer to non-intoxicant *C. sativa* biotypes. *Cannabis* includes genetically different biotypes of both industrial hemp and marijuana (Cit). This classification is typically based on a threshold concentration of cannabinoids. Cannabidiol (CBD) and Δ^9^-tetrahydrocannabinol (Δ^9^-THC) stand out as the most abundant and well-known cannabinoids present in this species. Δ^9^-THC predominates in marijuana, while CBD is more prevalent in industrial hemp. Industrial hemp, with its low THC content (0.5–0.2% *w*/*w*) in favour of CBD, is gaining prominence in scientific research. Additionally, industrial hemp is known for its ability to capture environmental CO_2_, remediate heavy metal-contaminated soils, and provide organic carbon, holding significant ecological value [14]. While fibres and seeds remain the primary products from the industrial variant, there is growing interest in exploring secondary metabolites such as terpenes, terpenophenols, amino acids, protein hydrolysates, fatty acids, and polyphenols [15].

*Cannabis* extracts are a central area of research focus [16,17,18]. Various extraction methods are being employed, with dynamic maceration standing out as a prominent approach. In dynamic maceration, alcoholic solvents are used, and the plant material is immersed in the solvent. This process efficiently releases bioactive components within the same solvent [19], demonstrating both antimicrobial and cellular effects.

Traditionally, cannabinoids are extracted using organic solvents, such as hydrocarbons (e.g., hexane) and alcohols (e.g., ethanol, methanol) [20,21,22]. While this method is cost-effective, easy to implement, and does not require complex equipment, it comes with significant drawbacks. The solvents used are flammable, toxic, and non-biodegradable, posing risks to human health and causing substantial environmental impact. Although efficient, solvent-based extraction can affect regulatory compliance and necessitate additional testing. For instance, residual solvent levels are strictly regulated for medicines produced under good manufacturing practices. Due to their toxicity, environmental hazards, and flammability, these solvents are less desirable for large-scale extractions.

In response to these challenges, researchers have explored alternative approaches based on green chemistry [23,24]. The pursuit of methods with high extraction yields, cost-effectiveness, scalability, and environmental friendliness remains a priority. Despite limited discussion in the literature, one study investigated extracts obtained from female inflorescences using water in conjunction with ultrasonic-assisted extraction (UAE). The results indicated that these aqueous extracts exhibit both antioxidant and anti-inflammatory effects, along with antimicrobial properties against *Candida albicans* [15]. The World Health Organization (WHO) has recently recognized the significant role of fungi in human health, as demonstrated by their recent publication of priority fungal pathogens [25]. It is estimated that fungal diseases affect over 1 billion individuals annually, leading to approximately 1.5 million deaths [26]. Notably, *Candida* species contribute significantly to this burden by also forming a biofilm on mucosal surfaces and adhering to biomedical implants or adjacent tissue. The emergence of new antifungal treatments has brought renewed optimism for managing fungal infections [27]. However, the number of antifungal drugs approved by the FDA remains limited. Given the significant tolerance of *Candida* spp. to conventional antiseptics and antifungal agents [28,29], alternative strategies are being explored, including the utilization of natural compounds from plant essential oils [30] and extracts [31,32,33].

Building upon this research direction and guided by the principles of the Green Circular Economy (GCE), this study explored the possibility of using waste from the light cannabis industry to produce bioactive extracts using water as the sole solvent. These extracts were tested for their antimicrobial activity on both planktonic and sessile cells of pathogenic strains of the *Candida albicans*, *Candida parapsilosis,* and *Candida tropicalis* species and for their antioxidant activity on HT-29 colon cancer cells under oxidative stress.

## 2. Results

### 2.1. HWEs Chemo-Physical Characterization

At the end of the short- and long-term maceration process, five different HWEs were obtained, as reported in Table 1.

All HWEs were firstly characterized for their chemical and physical properties by measuring their conductivity (μS cm^−1^), pH, fresh and dry weights (g), percentage of dry matter, and protein content (mg mL^−1^). As reported in Table 2, the characteristics of the HWEs diverged according to the raw material, the time of maceration, and the procedure followed in the extraction process (Table 2). The S21 extract exhibited the highest conductivity (10,500 μS cm^−1^), followed by S9 and S8 with 9003 and 4870 μS cm^−1^, respectively. S2 and S14 were the least conductive HWEs, with values of 205 μS cm^−1^ (S2) and 138.8 μS cm^−1^ (S14). The pH values for S21, S8, and S2 were slightly basic, while S9 and S14 were slightly acidic. The percentage of dry matter, calculated as the ratio between fresh and dry weight, ranged from 0.3% (S8) to 1.3% (S9). The quantification of the protein content was performed using the Bradford assay. HWEs obtained pre-treating the waste material using the microwave energy (MWE)-assisted process before the mechanical extraction (S2 and S8), and that obtained after one month of maceration (S9) displayed the lower protein content (0.43, 0.72, 0.55, respectively). Interestingly, the extract S21, obtained after 2 months of maceration, showed the highest protein content (5.7 mg mL^−1^), even higher than that of S14, the sole extract subjected to protein precipitation (2.9 mg mL^−1^).

### 2.2. HWEs Chemical NMR Characterization

All samples were prepared from lyophilized powder and dissolved in D_2_O. A comparison of the spectra recorded in D_2_O using the ZG pulse program (Figure S1) and the AU_WATERSC pulse program (Figure S2) clearly demonstrated that the latter allows for a clearer interpretation of the spectral region close to 4.8 ppm without any evident perturbation of the nearby signals. Hence, for the subsequent discussion of results, only the data obtained under water suppression conditions will be taken into consideration and some assignments were attempted by the comparison with the resonance data reported in the literature and in some public databases.

In order to investigate the presence of cannabinoids (that are not soluble in water), a defined volume of CDCl_3_ was added to the previously prepared sample, and the proton NMR spectra were recorded also in this solvent. Data collected in Figure S3 shows that even in an organic solution, the presence of cannabinoids is not detectable by NMR, as expected.

**Figure 1:**
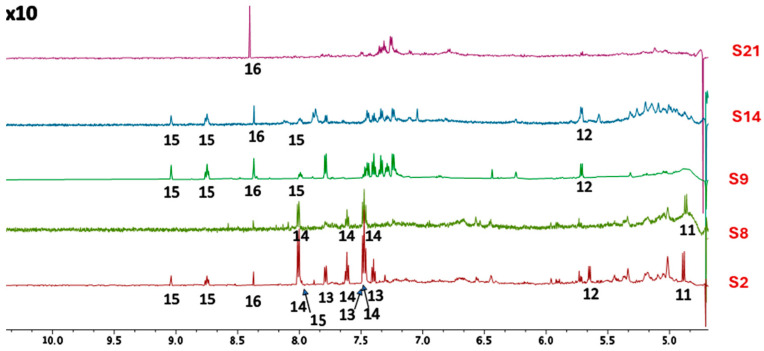
Low-field resonances (tenfold amplified) for spectra of samples S2, S8, S9, S14, and S21 were acquired in D_2_O using the AU_WATERSC pulse program. (11) Beta-glucose [34], (12) sucrose [34], (13) benzoic acid [35], (14) benzoyl derivatives (e.g., benzamide, or other amides), (15) trigonelline [34], (16) formic acid [34].

The five HWEs that were characterized underwent three different extraction methods: S2 and 8 were subjected to mechanical extraction, followed by microwave treatment, S14 to mechanical extraction and protein precipitation, and S9 and S21 were obtained only by long maceration and mechanical extraction. In the region from 4,5 ppm to 9,5 ppm (Figure 1), the samples treated with microwave energy (MWE) (S2 and S8) are unique because beta-glucose (11) [34] is present, probably arising from MW-assisted hydrolysis of sucrose (12) [34], which is present in S9 and slightly in S21 because the extracts were subjected only to microbial degradation. Sucrose is also present in extract S14, which underwent mechanical extraction and protein precipitation.

Among these two groups of samples, there is also another difference in the aromatic region. The MWE-treated group shows the presence of not fully identified benzoyl derivatives (14) (e.g., benzoyl amides) [34], while in the group treated with microbial degradation, there is no evidence of benzoic acid and its derivatives; but other different aromatic compounds can be identified at lower chemical shifts. In HWE S2 is evident the presence of benzoic acid (13) [36], which is not present in HWE S8 which belongs to the same group of treatment but differs for the raw material used.

The presence of trigonelline (15) is noticeable in S2, S9, and S14, while formic acid (16) is present in S2, S9, S14, and S21 (while in S8 it was only present in traces).

**Figure 2:**
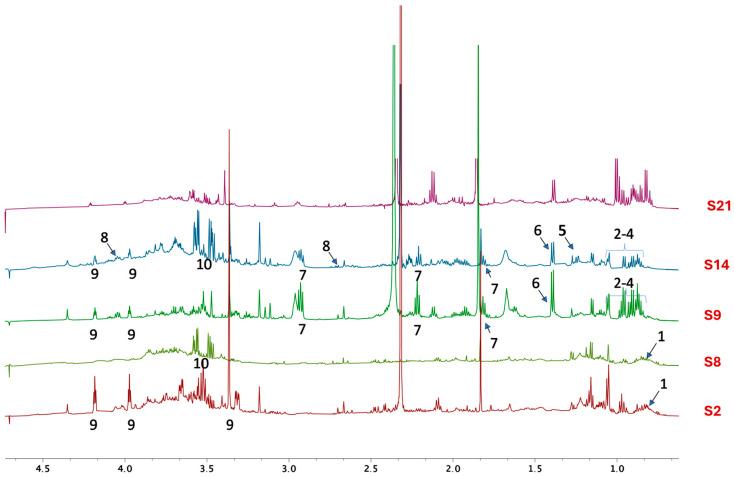
High-field resonances for spectra of samples S2, S8, S9, S14, and S21 were acquired in D_2_O using the AU_WATERSC pulse program. (1) Fatty acids [37], (2) valine [34,38], (3) isoleucine [34,39], (4) leucine [34,40], (5) lactate [34,41], (6) alanine [34], (7) GABA [42], (8) malic acid [43], (9) choline [34], (10) glycerol and its derivatives [44].

At low-field frequencies, S14, the only HWE treated with protein precipitation, shows a profile similar to that observed for S9 (Figure 2). In the spectral region between 0 ppm to 4.5 ppm, high-field resonances can detect the same clusters of similarities/differences. S2, S9, and S21 (but not S8 and S14) showed coupling patterns associable to choline (9). In S9, S21, S8, and S14 are evident signals that can be assigned to valine (2), isoleucine (3), leucine (4), and alanine (6).

S14 was the only one that showed a minor content of malic acid (8) and lactate (5), while GABA (7) was observed only in S9 and S14. A non-clear distribution was observed for glycerol/glycerol derivatives (10) (detected in samples S8 and S14), while fatty acids (1) [37] were detected in all the samples.

### 2.3. Antifungal Susceptibility Testing

**Figure 3:**
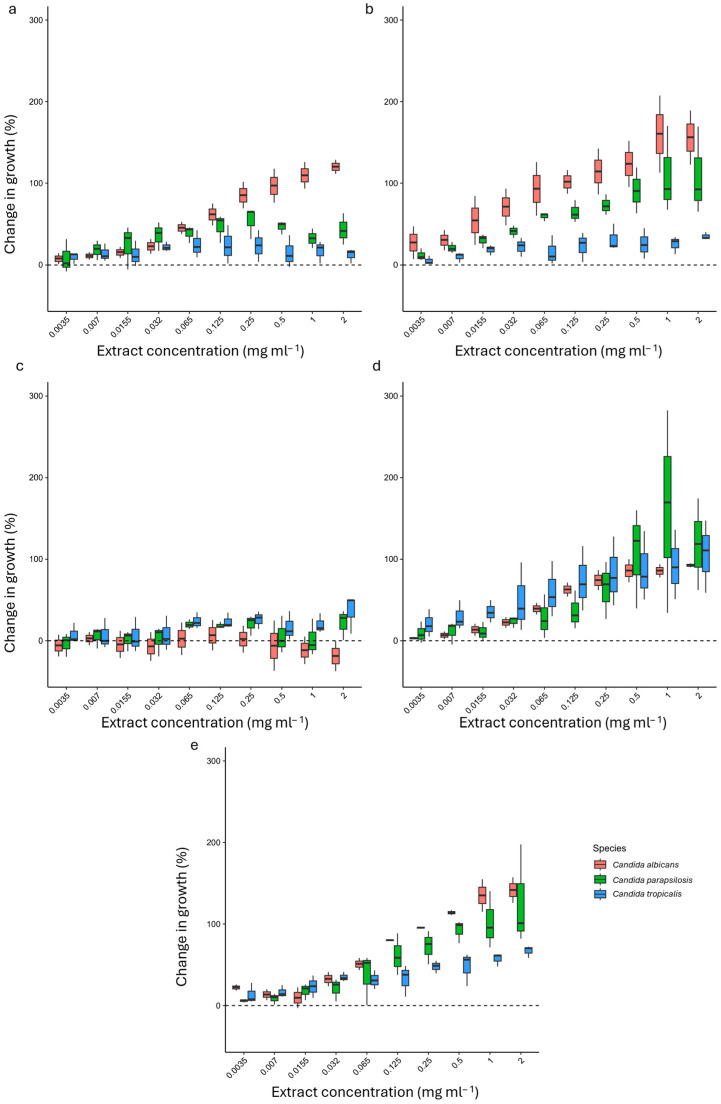
Effect of HWEs on *Candida* planktonic growth after 24 h (HWE 2 in panel (**a**), 8 in panel (**b**), HWEs 14 and 21 in panels (**c**) and (**d**), respectively, and HWE 9 in panel (**e**). The HWEs were added in the medium of growth at different concentrations that ranged from 2.0 mg mL^−1^ to 0.0035 mg mL^−1^. Values are expressed as percentage variation with respect to the control. Positive values indicate an increase, while negative values indicate a decrease in yeast biofilm growth.

The impact of all HWEs on the growth of eight strains of *C. albicans*, *C. parapsilosis*, and *C. tropicalis* (CA1, CA2, CT1, CT2, CT3, CP1, CP2, and CP3) was investigated across a range of concentrations from 2 mg mL^−1^ to 0.0035 mg mL^−1^. The results, expressed as the percentage change in growth compared to the control, are summarized in Figure 3 and Supplementary Table S1.

The addition of the HWEs stimulated the growth of the eight strains tested with a dose-dependent response. Overall, the lower concentration boosted the growth of planktonic cells to a lesser extent than their higher counterpart.

Extracts S2, S8 (from short-term macerates, Figure 3a,b), and S9 (from one month macerate, Figure 3e) mostly exhibited their enhancing effects on both strains of *C. albicans*, as well as on strain *C. parapsilosis* (CP3) (S8 and S9). Conversely, S21 (from two months macerate (Figure 3d) consistently enhanced the growth of all strains, reaching its peak influence on strain CP3 (*C. parapsilosis*), where a 413.6% increase was observed at a 2 mg mL^−1^ concentration.

Interestingly, only S14 (Figure 3c) exhibited a significant inhibitory effect on the growth of the CA1 *C. albicans* strain across all tested concentrations, ranging from −5% at 0.007 mg mL^−1^ to −37% at 2 mg mL^−1^. Also, the *C. tropicalis* (CT3) strain was inhibited at low concentrations of this extract (ranging from 0.0035 to 0.032 mg mL^−1^), while the *C. parapsilosis* strain (CP2) displayed reduced growth across all concentrations except those within the range of 0.065 to 0.25 mg mL^−1^. Finally, an inverse growth trend was observed for strains CA2 and CT1, reaching their highest increase at 0.25 mg mL^−1^ and decreasing to a minimum at the highest concentration of 2 mg mL^−1^.

### 2.4. Influence of HWEs on Biofilm Adhesion and Biofilm Development

All strains employed to evaluate the impact of the HWEs on planktonic growth were able to form biofilm in vitro [45]. The HWEs were tested for their potential to influence both biofilm adhesion and biofilm development (Supplementary Tables S2 and S3, respectively) at the same concentrations tested for the assays of planktonic growth.

**Figure 4:**
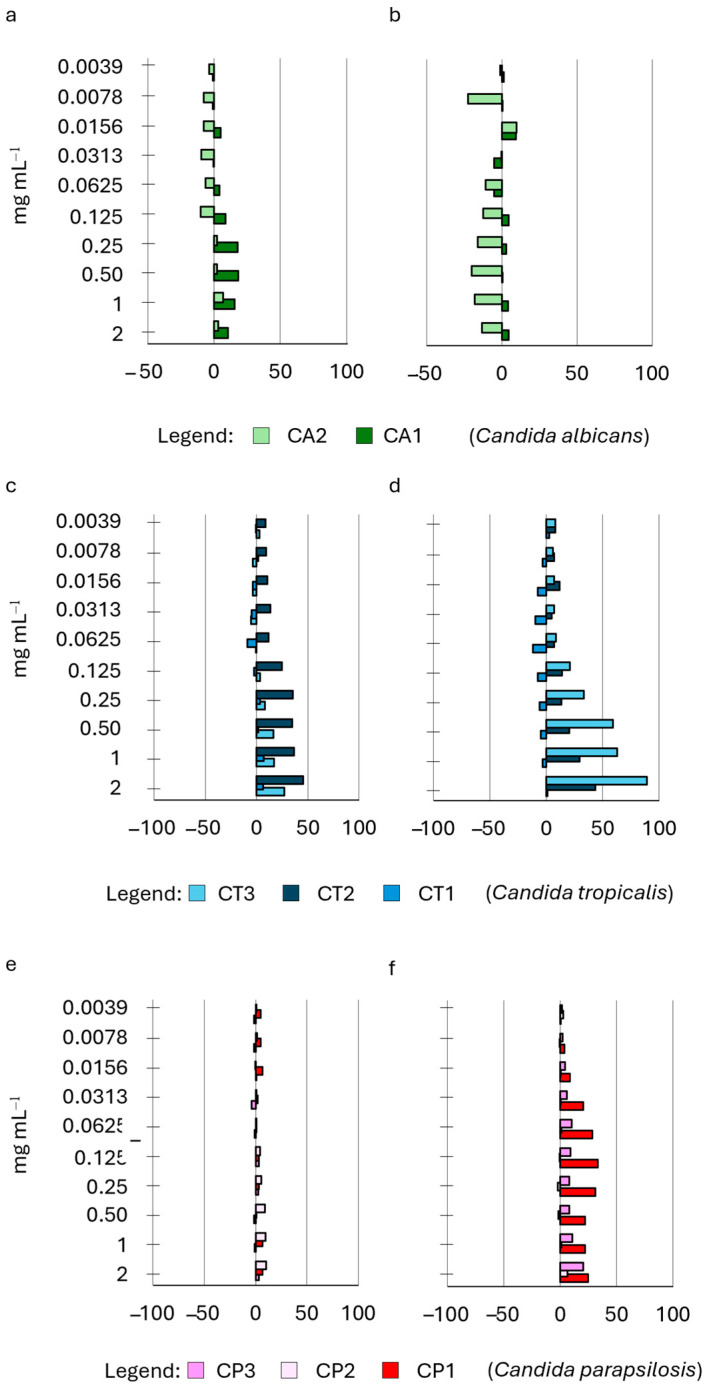
Effect of HWE S14 on *Candida* biofilm growth at 24 h. The HWE was added during either the priming phase of biofilm (panels (**a**,**c**,**e**)) or the subsequent development phase (panels (**b**,**d**,**f**)). The concentrations of the HWE ranged from 2.0 mg mL^−1^ to 0.0035 mg mL^−1^. Values are expressed as percentage variation with respect to the control. Positive values indicate an increase, while negative values indicate a decrease in yeast biofilm growth.

The efficacy of S14 HWE in inhibiting biofilm adhesion and formation was comparatively minimal (Figure 4). The strains exhibited a limited correlation between the biofilm produced when the extract was applied during the adhesion phase and when it was exclusively used in the biofilm development phase. Specifically, when S14 was employed during the adhesion phase of biofilm formation, it demonstrated a modest negative impact on the biofilm developed after 24 h (CA1, CA2, CT1, and CT2, Figure 4a,c) predominantly at lower concentrations (0.0039–0.125 mg mL^−1^). When S14 was exclusively applied during the biofilm development phase, it showed a negative impact, reaching a maximum value of –20% and particularly affecting two strains (CA2 and CT1, Figure 4b,d) across almost all tested concentrations with varying degrees of decrease.

**Figure 5:**
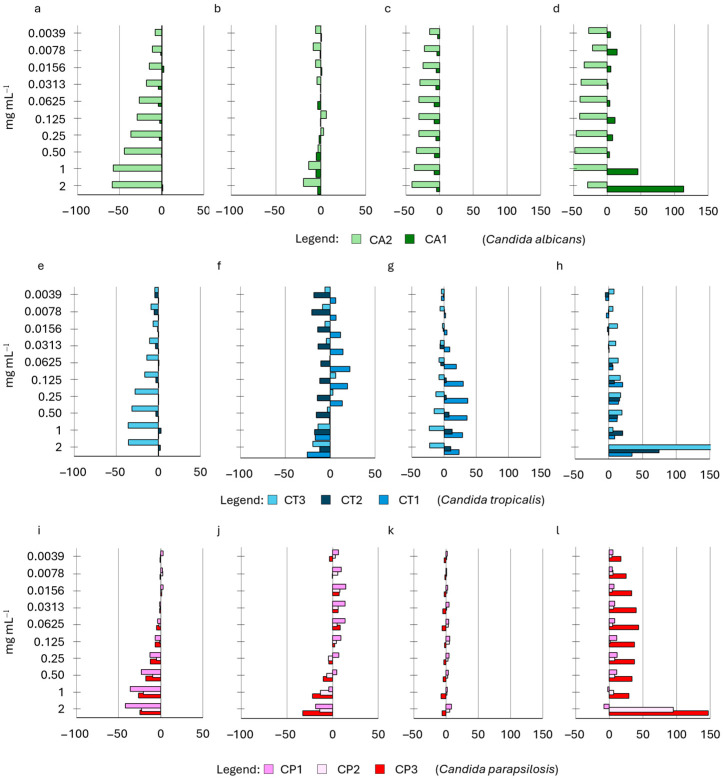
Effect of HWE S2 and S8 on *Candida* biofilm growth at 24 h. The HWEs were added during either the priming phase of biofilm ((panels (**a**,**e**,**i**) for HWE S2 and panels (**c**,**g**,**k**) for HWE S8) or the subsequent development phase (panels (**b**,**f**,**j**) for HWE S2 and panels (**d**,**h**,**l**) for HWE S8). The concentrations of the HWE ranged from 2.0 mg mL^−1^ to 0.0035 mg mL^−1^. Values are expressed as percentage variation with respect to the control. Positive values indicate an increase, while negative values indicate a decrease in yeast biofilm growth.

We examined the impact of HWE S14 on the growth of the *Candida* strains’ biofilm at ten different concentrations, comparing it to the control. Notably, the HWE was administered exclusively during either the priming phase of biofilm development or the subsequent development phase. HWEs S2 and S8, obtained using the same extraction methods but from distinct raw materials (dry and fresh plants), exhibited varying effects on biofilm adhesion and development (Figure 5).

**Figure 6:**
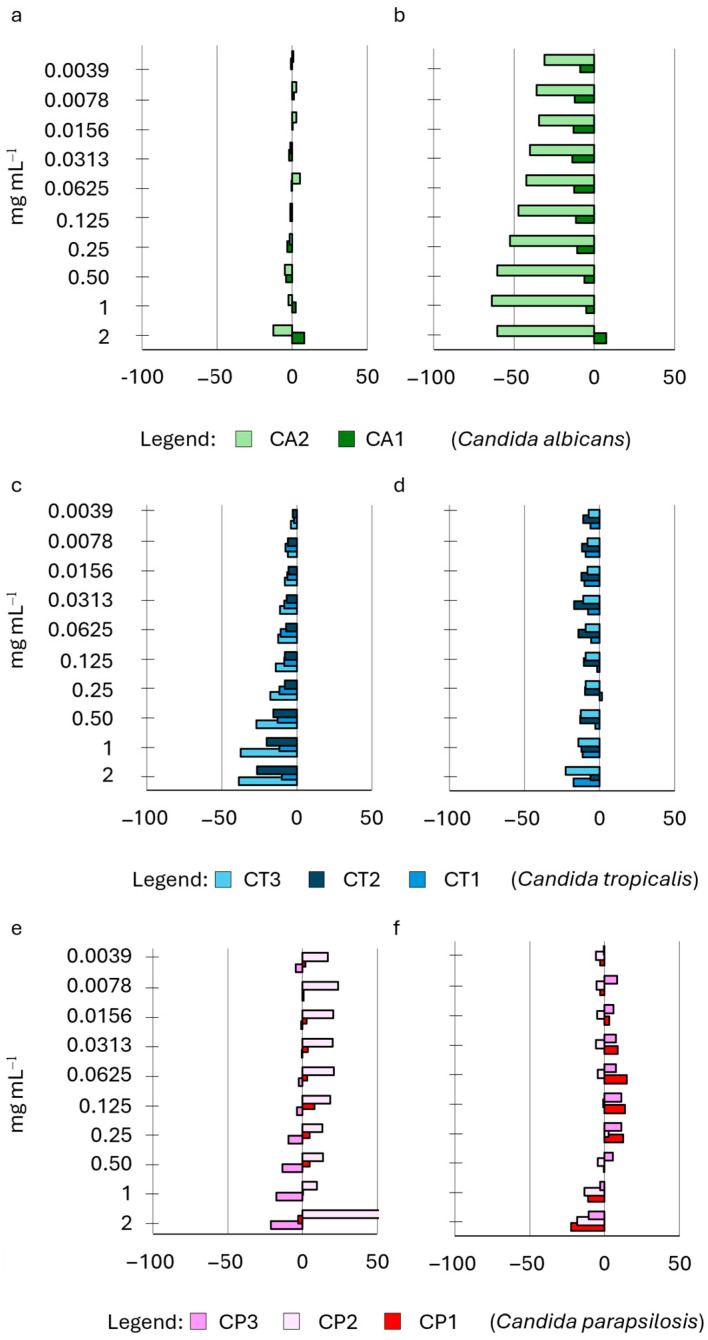
Effect of HWE S9 on *Candida* biofilm growth at 24 h. The HWE was added during either the priming phase of biofilm (panels (**a**,**c**,**e**)) or the subsequent development phase (panels (**b**,**d**,**f**)). The concentrations of the HWE ranged from 2.0 mg mL^−1^ to 0.0035 mg mL^−1^. Values are expressed as percentage variation with respect to the control. Positive values indicate an increase, while negative values indicate a decrease in yeast biofilm growth.

Interestingly, both HWEs demonstrated more favourable outcomes when applied during the adhesion phase rather than the development phase (Figure 5a,e,j for S2 and Figure 5c,g,k for S8). When applied exclusively during the adhesion phase, HWE S8 exhibited a reduction in the quantity of biofilm produced by five strains (CA2, CT3, CP1, CP2, and CP3, Figure 5c,g,k), showing varying degrees of decrease. The observed effects were consistently influenced by concentration, ranging from an average of −2% at 0.003 mg mL^−1^ to an average of −37% at 2 mg mL^−1^. The remaining three strains were either unaffected by the HWE (CT1) or experienced negative effects but in a non-concentration-dependent manner. When administered during the biofilm development phase, extract S8 exhibited a considerably milder effect at low concentrations, demonstrating a boosting effect on biofilm development instead (Figure 5d,h,i). Notably, only one strain, CT2, experienced consistent inhibition across all concentrations, while the others showed a consistent decrease in biofilm formation from 0.25 to 2 mg mL^−1^. The impact of extract S2 on biofilm adhesion was notably less pronounced compared to extract S8 as it strongly inhibited the adhesion of only two strains (CT3 and CA2). The remaining strains were either minimally affected (CP1 and CA1) or experienced a boost. Strain CA2 was the only one influenced by the application of S2 during the biofilm development phase, with the other strains experiencing a concentration-dependent enhancement (Figure 5d). Conversely from the previously mentioned HWEs, S9 and S21 (derived from long maceration and mechanical extraction only) exhibited a more pronounced average inhibition when applied during the biofilm development phase rather than the biofilm adhesion phase. Both HWEs demonstrated a distinct consistent overall trend when considering all the strains together. Specifically, biofilm formation was inhibited by S9 (Figure 6b,d,f), averaging at −10% from 0.003 to 0.03 mg mL^−1^, with a decreasing impact that reached its minimum at 0.25 mg mL-1 and increasing again from 0.5 to 2 mg mL^−1^.

**Figure 7:**
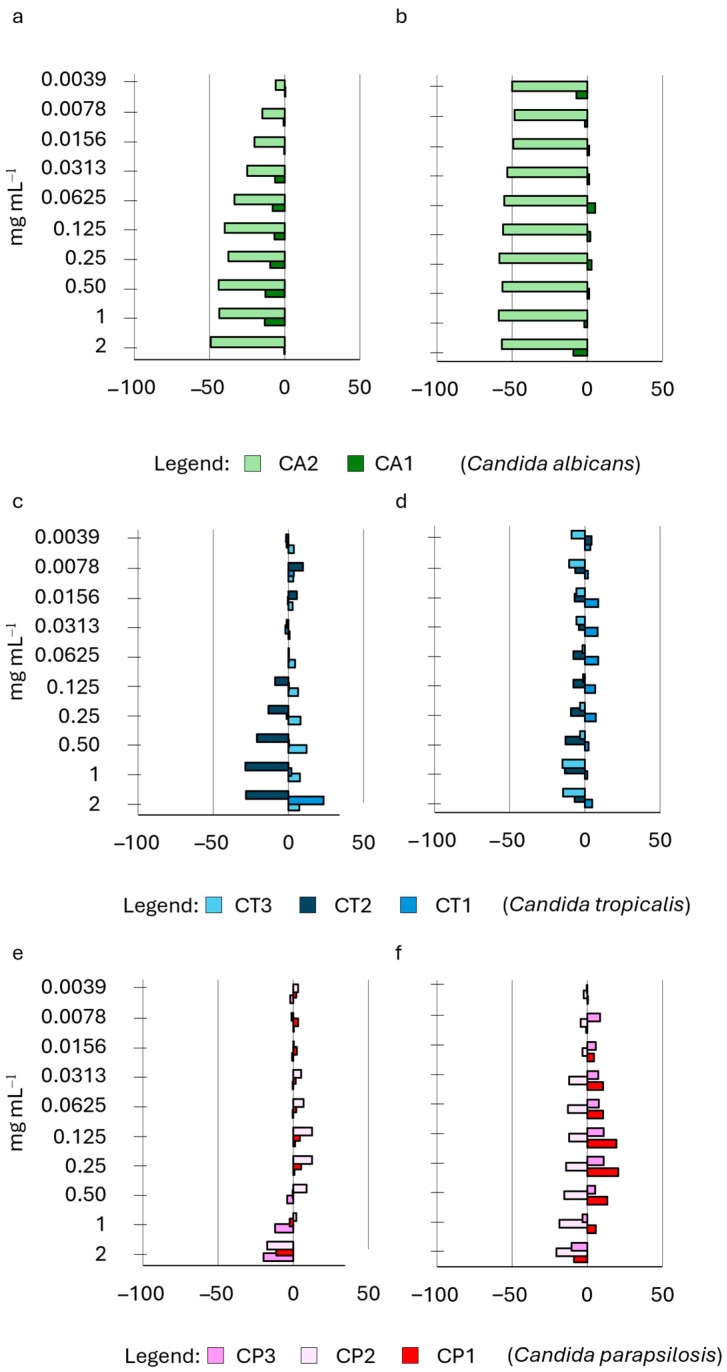
Effect of HWE S21 on *Candida* biofilm growth at 24 h. The HWE was added during either the priming phase of biofilm (panels (**a**,**c**,**e**)) or the subsequent development phase (panels (**b**,**d**,**f**)). The concentrations of the HWE ranged from 2.0 mg mL^−1^ to 0.0035 mg mL^−1^. Values are expressed as percentage variation with respect to the control. Positive values indicate an increase, while negative values indicate a decrease in yeast biofilm growth.

A comparable effect was observed with HWE S21 (Figure 7b,d,f), displaying a similar trend of inhibiting biofilm formation when applied during the biofilm development phase. When applied during biofilm adhesion, HWE S9 (Figure 7) inhibited cell adhesion for all strains but CP3, CP2, and CA1, while HWE S21 exerted a less coherent action on the strains used.

The reduction of cell adhesion was null for strains CT1 and CT2 which were boosted at higher concentrations. On the other hand, strains CA2, CT3, CP1, and CP2 were inhibited, resulting in a negative impact on the biofilm adhesion phase with varying degrees of reduction in a concentration-dependent manner. Finally, CT2 was boosted at the highest concentration tested, and CP3 was boosted at the central concentrations and inhibited at 2 mg mL^−1^.

### 2.5. Antioxidant Activity of HWEs on HT-29 Colon Cancer Cells

**Figure 8:**
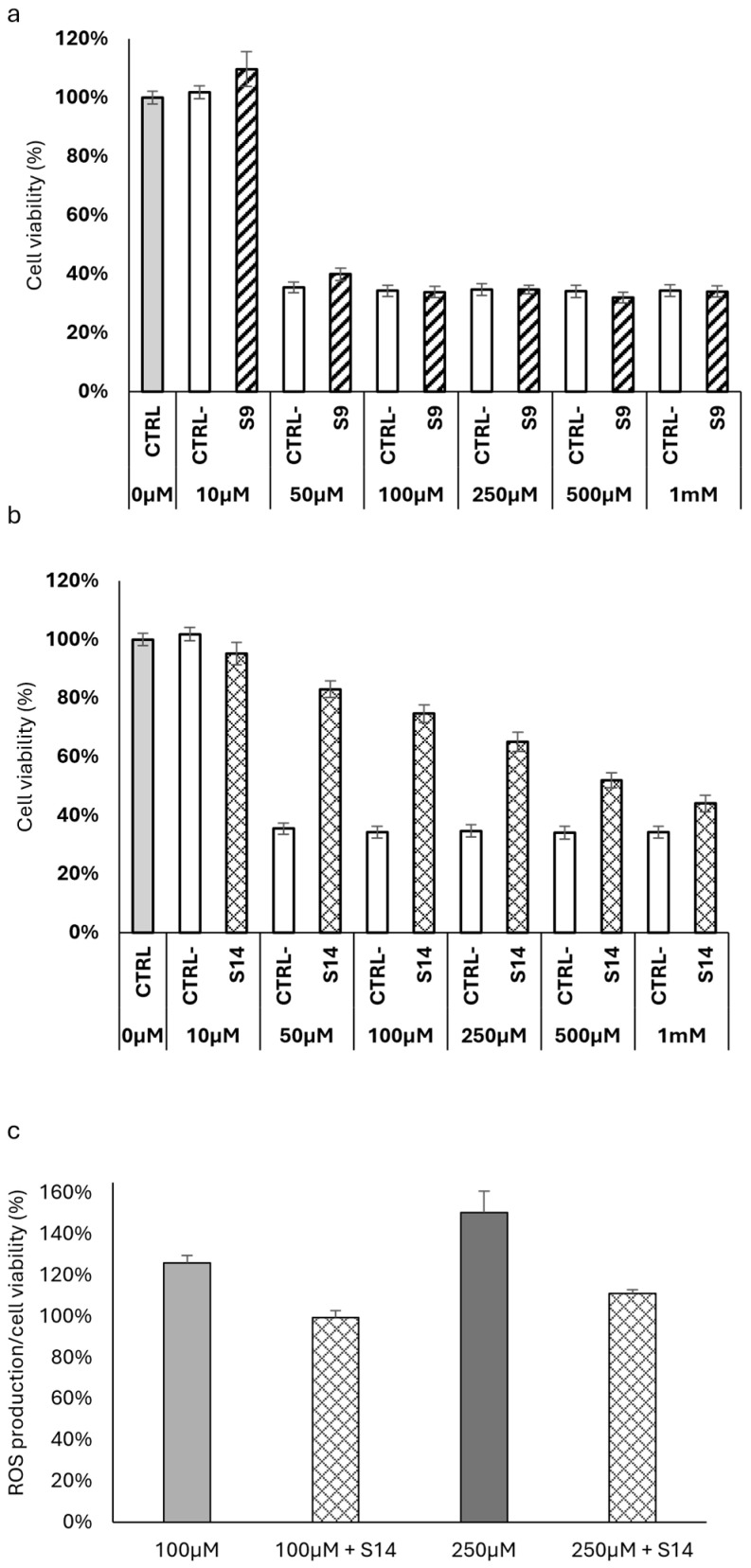
Assessment of the cellular viability of HT-29 colon cancer cells under oxidative stress induced by H_2_O_2_, both with and without the same concentration of S9 (panel (**a**)) and S14 (panel (**b**)). The positive control corresponds to cell viability under normal conditions without oxidative stress, whereas the negative control reflects cell viability resulting from oxidative stress induced by varying concentrations of H_2_O_2_. Scavenging activity of S14 against ROS production induced by 100 µM and 250 µM of H_2_O_2_ (panel (**c**)).

In this study, hydrogen peroxide was employed to induce oxidative stress, given its status as one of the most commonly produced reactive oxygen species (ROS). Oxidative stress is characterized by an imbalance between ROS production and the antioxidant defence system and plays a critical role in the development of various diseases, including cancer. Antioxidants derived from natural sources, such as plant HWEs, have gained attention for their potential to counteract oxidative stress-induced damage. This study aimed to investigate the antioxidant effects of S9 and S14 HWEs on HT-29 colon cancer cells under oxidative stress induced by H_2_O_2_. The experimental results revealed contrasting effects of S9 and S14 HWEs on HT-29 cell viability following exposure to different concentrations of H_2_O_2_, emphasizing how the preparation process of these HWEs plays a crucial role in preserving molecules with pronounced antioxidant activity. While S14 (Figure 8b) demonstrated a significant antioxidant effect across all H_2_O_2_ concentrations tested, S9 (Figure 8a) did not exhibit a protective effect on the cell line under oxidative stress conditions. This effect became particularly evident starting from the concentration of 50 µM of H_2_O_2_, which resulted in more than 60% cell mortality. However, in the presence of S14 (Figure 8b), cellular vitality exceeds 80%, indicating a potent antioxidant effect of the HWE. This effect is also observed at higher concentrations of hydrogen peroxide, although with increasing concentration of the oxidizing agent, the protective effect becomes less pronounced but still statistically significant.

The origin of the observed antioxidant activity of S14 toward H_2_O_2_ was also investigated through experiments aimed at searching for reactive oxygen species (ROS). As shown in Figure 8c, S14 has demonstrated an interesting scavenging activity against the two concentrations of H_2_O_2_, inducing a decrease in fluorescence intensity after the treatment with DCFH-DA.

## 3. Discussion

The increase in the cultivation of industrial hemp (*Cannabis sativa* L.) and the different possibilities of use generate useful by-products along the entire industrial chain [46,47]. In this context, the aim of this study was to verify the possibility of recycling industrial hemp processing waste to produce water extracts rich in bioactive compounds, following a simple, cheap, and green extraction protocol, according to the basic principles of Green Circular Economy (GCE). The extracts were obtained using simple mechanical pressing at different times of maceration of the waste in water, which also verified the effectiveness of the MWE-assisted protocol in the extraction of the compounds from the raw material. This approach led to the production of five different water extracts (S2, S8, S14, S9, and S21). All extracts were characterized for their chemo-physical and biological properties, specifically testing their antioxidant, antifungal, and anti-biofilm activity, in light of their possible use for different biomedical applications.

HWEs S2 and S8 were obtained with the same procedure (72 h maceration followed by mechanical extraction and MWE-assisted process) with the only difference residing in the use of dry (S2) and fresh (S8) waste. This impacted their chemical profile (Figure 1 and Figure 2) by being S8 rich in free amino acids and glycerol while being S2 rich in benzoic acid, trigonelline, formic acid, and choline. If free amino acids are known to have no influence on the biofilm formation and development phase [48], the other compounds are known to interact negatively with biofilm formation [49,50,51]. Despite their different composition, neither showed any inhibitory activity on the growth of planktonic cells, with no observable MIC (Minimum Inhibitory Concentration) values. On the contrary, both HWEs negatively affected biofilm adhesion, while they showed an opposite effect on biofilm development with S2 acting as a booster and S8 depressing the amount of biofilm formed. As expected, these results underline how the choice of raw material modifies the chemical profile and, consequently, the biological properties of the extract. However, from both the dry residue and the fresh residue, it was possible to obtain extracts rich in useful compounds, highlighting how both approaches could be pursued to generate new bio-based value chains in sustainable innovation for biomedical applications [52,53]. HWEs S9 and S21, which followed the same extraction protocol but differed in maceration duration (1 and 2 months, respectively), exhibited remarkably similar profiles. The only difference between the two extracts was the presence of trigonelline and GABA in S9, absent in S21. Despite this, both HWEs demonstrated a strong inhibitory activity in biofilm formation when applied to the development phase, while a weaker effect when applied only during the adhesion phase. Although further research is needed to clarify their specific mechanism of action, these results suggest that the length of the maceration period heavily influenced their chemical composition, favouring the extraction of antimicrobial compounds otherwise not present. Further studies will therefore be necessary to understand how the duration of maceration influences the quality of the extracts focusing on the role of the native microbial community in the entire process [54,55].

Out of all HWEs, S14 exhibited the most diverse and complex chemical profile being the only one that underwent protein precipitation. Notably, it was the sole extract containing both malic acid and lactate, already recognized for their anti-biofilm activities [48,56]. This compound exhibited contrasting effects on the *Candida* strains being the only one to have negative interaction with planktonic growth but, on the other hand, acted as a strong booster for biofilm adhesion and development. Although none of the effects of these extracts could be directly attributed to a specific class of molecules unique to each extract, whether their behaviour and interactions with cells can be attributed to their overall complexity and the potential interactions among the compounds present is a topic for future research. Moreover, S14 exerted its antioxidant effect on hydrogen peroxide-stressed cells which behaviour may be attributed to the presence of bioactive compounds within cannabis, and this result was also confirmed by the scavenging activity against ROS produced by the treatment with different concentrations of H_2_O_2_. As emerged from the NMR analyses (Figure 1 and Figure 2), S14 HWE is rich in valine, isoleucine, leucine, lactate, alanine, GABA, and malic acid. Recent research has shed light on the potential antioxidant properties of several compounds, including branched-chain amino acids (BCAAs) such as valine, isoleucine, and leucine. While primarily recognized for their roles in protein synthesis and muscle metabolism, emerging evidence suggests that BCAAs may exert antioxidant effects through various metabolic pathways [57]. Furthermore, the neurotransmitter γ-aminobutyric acid (GABA) has garnered attention for its potential antioxidative role, attributed to its modulation of specific GABA receptors within the brain [58]. Furthermore, researchers have explored the antioxidant potential of malic acid [58,59], a compound exclusively found in HWE S14. Malic acid serves as a crucial intermediate in the Krebs cycle and exhibits electron-donating properties within the electron transport chain. As a result, it effectively reduces the production of free radicals. Collectively, these findings highlight the multifaceted nature of these compounds, underscoring their potential as antioxidants within various physiological contexts.

## 4. Materials and Methods

### 4.1. Hemp Waste 

Waste of *Cannabis sativa* L., including flowers, fibres, and leaves, was kindly supplied by JJ Farm Società Agricola Semplice (Castiglione del Lago (Pg), Umbria, Italy). The study was conducted using the Strawberry variety, cultivated in an outdoor natural condition without any addition of chemicals. The plants were harvested manually and immediately dried in closed sheds, in the dark, and using ventilated ovens to facilitate the drying process. The flowers were then separated from leaves and stems using a mechanical system, and the waste (stems, leaves, and floral residues) was collected in sealed plastic bags and stored in the dark, at room temperature, until further analyses. 

### 4.2. Extraction Procedure 

Wastes were gently crushed into a small size to facilitate the extraction procedure. To evaluate whether, and to what extent, the time of the maceration process influences the chemical, physical, and biological properties of the Hemp Water Extracts (HWEs) obtained, two different protocols (short- and long-term maceration processes) were optimized.

#### 4.2.1. Short-Term Maceration Process

Hemp waste has been divided into two groups: (i) dried stems, leaves, and floral residues and (ii) fresh stems, leaves, and floral residues recovered before the drying process. For each thesis, one hundred grams of residues were placed in a sterile glass jar and covered with 800 mL of sterile deionized water at room temperature (approximately 25 °C). Each suspension was then mixed and left to macerate for 72 h in the dark at 25 °C. Three biological replicas were prepared, each tested in triplicate. At the end of maceration, the liquid and solid fractions were separated and subjected to the extraction process using mechanical pressing. The final solid residue was collected and stored for further analysis. Each extract was then sterilized by filtration using Büchner filters (1.2 µm up to 0.2 µm diameters, Sigma-Aldrich, St. Louis, MO, USA) to guarantee the sterile condition and subjected to the microwave energy (MWE)-assisted process for 3 min at 500 W. A volume of 100 mL was then taken for the application of the protein precipitation protocol (as detailed in Section 4.2.2). All sterile extracts were freeze-dried and stored at −80 °C before further analysis.

#### 4.2.2. Protein Precipitation

For the precipitation of the protein fraction, ethanol was used as a precipitation system as reported in the work of Koh et al. [60], with some modifications. Briefly, 10 mL of the macerate obtained from the dry waste, after sterilization by filtration, was treated by adding Potassium Acetate 3 M in a volume equal to 1/10 of the sample volume, and cold absolute ethanol was used at twice the volume of the sample. The sample was then shaken and left for 20 min at −20 °C and then centrifuged at 12,000× *g* for 15 min at 4 °C. The supernatant was recovered and lyophilized while the pellet was discarded. 

#### 4.2.3. Long-Term Maceration Process

One hundred grams of dried leaves, inflorescences, and stems were gently crushed into small sizes to facilitate the extraction procedure and placed in a sterile glass jar covered with 800 mL of sterile deionized water. Two vases were obtained with the same procedure, one was left macerating for 1 month while the other was for 2 months. Both extracts were macerated in the same condition of temperature (25 °C). At the end of the maceration, both samples were mechanically processed to separate the liquid fraction from the solid part. The liquid fractions were filtered using Büchner filters (1.2 µm and after 0.2 µm) to guarantee a sterile condition.

### 4.3. Hemp Water Extracts (HWEs)

At the end of the short- and long-term extraction processes, five different HWEs were obtained, as detailed in Table 1.

### 4.4. HWEs Chemo-Physical Characterization

#### 4.4.1. PH, Electrical Conductivity (EC) and Salt Concentration 

The pH measurements for all the HWEs obtained by water extraction were performed using the instrument pH 50VioLab (Dostmann Gmbh, Wertheim-Reicholzheim Germany), Bench Meter. All the measurements were performed in triplicate.

The electrical conductivity of the extracts was measured with a conductivity EC-meter CRISON-GLP-31 (Crison Instruments S.A., Alella, Spain) at 25.0 ± 0.1 °C. The final value was converted to a standard ECe parameter using the conversion factor proposed by Lee et al. [61].

#### 4.4.2. Dry Weight

At the end of the mechanical extraction, all the HWEs were studied with the final purpose of determining the dry weight residual after the procedure. Briefly, 1mL of each extract was loaded into a thermobalance and the fresh weight was calculated. After that, the samples were warmed up at a constant temperature of 105 °C until obtaining a constant weight value. All the measures were made in triplicate. 

#### 4.4.3. Protein Quantification by Bradford Assay

All the HWEs were also tested to determine protein content using a Bradford assay. The Bradford assay was performed according to Marion M. Bradford using Quick Start™ Bradford 1× Dye Reagent (Bio-Rad, Hercules, CA, USA) according to the manufacturer’s instructions for one-step determination of protein concentration. Briefly, the samples were diluted 1:10 in dd H_2_O to reach a standard concentration (*w*/*v*) of 1 mg mL^−1^. The assay was performed in 1 mL of final volume according to the instructions. The dye reagent was used 1:2 on the final volume and the remaining part was represented by dd H_2_O and sample. The quantitative determination was carried out using the Coomassie Brilliant Blue G250 dye (Bio-Rad, Hercules, CA, USA), which has an absorption peak of 595 nm in the protein-bound form. The absorbance was measured at 595 nm using a Shimadzu UV-160A UV-Visible Recording Spectrophotometer (Shimadzu Scientific Instruments, Kyoto, Japan). The protein content was determined by the absorbance value using a calibration curve obtained by well-known concentrations of Bovine Serum Albumin (BSA; Sigma-Aldrich, Saint Louis, MO, USA). The measurements were carried out in triplicates and the protein content is expressed in mg mL^−1^.

#### 4.4.4. NMR

NMR measurements were obtained at 25 °C on a Bruker spectrometer operating at 600 MHz (^1^H) and equipped with Cryoprobe Prodigy. Chemical shifts (δ) are reported in parts per million (ppm) and they are relative to TMS 0.0 ppm (for CDCl_3_) and indirectly to DSS using the residual signal of water at 4.65 ppm (for D_2_O). The samples were prepared dissolving from 5 to 15 mg of lyophilized powder in D_2_O and subsequently extracted in CDCl_3_ purchased by Sigma-Aldrich. All the spectra were obtained by processing the FIDs using MestReNova v14.3.1 and applying an automatic baseline correction and manipulation with an LB = 3 exponential function (EF). Spectra in water were acquired both with the normal ZG pulse program or using the program “au_watersc” for the acquisition of water-suppression spectra in automation.

### 4.5. Antimicrobial Activity Assay 

With the purpose of evaluating the potential cytotoxic activity against human pathogen *Candida*, the HWEs were used for an antifungal susceptibility test where the HWEs were tested at ten different concentrations starting from 2mg mL^−1^ until 0.0325 mg mL^−1^. The test was performed following the guidelines of EUCAST E.Def 7.3.2 [62]. All the HWEs were prepared at the stock concentration of 40 mg mL^−1^ using sterile deionized sterile water. The eight *Candida* strains that were used in the tests (Table 3) were isolated from clinical samples of different hospital structures located in Italy and conserved in a dedicated collection of the laboratory at −80 °C.

Before testing, each strain was cultured in agar plates of YAPD + Chloramphenicol at the concentration of 0.5 g/L and was left incubated at 37 °C for two days. At the end of incubation, one colony from each agar plate was used for pre-inoculum in 10 mL of the same liquid medium (YAPD + Chloramphenicol) and incubated overnight at 37 °C under shaking conditions (120 rpm). At the end of the growing period, the optical density was measured at 600 nm using a spectrophotometer instrument (Jasco V-730, Spectrophotometer, Jasco Europe Srl, Lecco, Italy), and the OD_600_ was standardized at the value of 0.2, two-fold the OD value used for the test. The cells were then resuspended in a liquid RPMI 1640 liquid medium (Sigma-Aldrich, St. Louis, MO, USA) added with glucose and MOPS (Sigma-Aldrich) at the concentrations of 36 g L^−1^ and 69,06 g L^−1^, respectively. All HWEs were prepared two-fold to the higher concentration used by diluting the stock solution 1:10 using deionized sterile water. In total, 100 μL of the highest concentration was added in all the wells of column number 1, and 50 μL was placed in all the other wells of the 96 multiwell plate. Using a multichannel pipette, the HWEs were diluted following the principle of microdilution in the plate, relocating 50 μL from column 1 to the others until column number 10. Column 11 was used as a positive control and column number 12 as the control of the medium and HWEs. Subsequently, 50 μL of the standardized cell suspensions were added in all columns but the last. The plates were then sealed with parafilm to avoid excessive evaporation and were incubated for 24 h at 37 °C. The day next, the volume of each well was resuspended and measured at 405 nm with a Sunrise plate reader. Afterwards, 30 μL of resazurin at a concentration of 0.015% was added to each well of the plates and left incubating at room temperature for 2–4 h. Before use, the resazurin solution was sterilized using filtration (0.22 filters) and stocked at 4 °C.

### 4.6. Biofilm Formation 

To determine the potential anti-biofilm activity exerted by the Hemp Water Extracts (HWEs), the biofilm assay was made in two different ways to determine if the HE can interfere with the adhesion or development of the biofilm, using the assay protocol of Christopher G. Pierce et al. [63], with some modifications [64,65]. Briefly, each *Candida* strain was grown in 10 mL of the appropriate medium (YEPD + Chloramphenicol) in a 100 mL bottle to maintain the appropriate head space for the oxygenation. The strains were left for 24 h at 37 °C in an orbital shaker at 150–180 rpm. After 24 h of incubation, the cells were resuspended in an RPMI-1640 (Sigma-Aldrich, Saint Louis, MO, USA) to obtain a cell density of OD_600_ = 0.2. After that, 50 µL of this standardized cell suspensions were seeded in selected wells of a 96-microtiter plate; cells without treatment were used as a positive control, and unseeded wells were considered as a negative control. To determine the effect of HWEs during the adhesion phase, 50 µL of the hemp HWEs were added in selected wells for a final concentration ranging from 2 mg mL^−1^ to 0.003 mg mL^−1^. The plates were incubated for 2 h at 37 °C and subsequently washed three times with a PBS solution. Finally, 100 μL of fresh medium was added to each well.

The second approach was that of testing the HWEs in the phase of biofilm development. The inoculum calibrated at OD_600_=0.1 was added to each well, and the plates were incubated at 37 °C for two hours and washed trice afterwards. Subsequently, 50 µL of fresh medium (2×) was added to each well along with 50 µL of each HE at twice the concentration for each tested concentration.

After 24 h of incubation at 37 °C, the plates were washed thrice with the PBS solution and were stained with 100 µL of Crystal violet solution at 0.1%. The plates were incubated for 15 min and then were washed three times with sterile water and dried at room temperature until measured at 570 nm in a Tecan plate reader (Tecan Infinite Nano+, Tecan Trading AG, Mannedorf, Switzerland).

### 4.7. HWEs Activity on HT-29 Colon Rectal Human Tumor Cell Line

Dulbecco’s modified Eagle’s medium (DMEM), fetal bovine serum (FBS), Trypsin, and Penicillin/Streptomycin were purchased from Euroclone (Pero, Italy). Dimethyl sulfoxide (DMSO), Trypan blue powder, hydrogen peroxide solution (35%), and 3-(4,5-dimethylthiazol-2-yl)-2,5-diphenyltetrazolium bromide (MTT) were purchased from Sigma-Aldrich (Saint Louis, MO, USA) and from Becton, Dickinson and Company (Franklin Lakes, NJ, USA).

#### 4.7.1. HT-29 Cell Culture

HT-29 human colorectal adenocarcinoma cells (ATCC, Manassas, VA, USA) were cultured in a DMEM medium containing 10% (*v*/*v*) heat-inactivated FBS and Penicillin 10,000 U per mL/Streptomycin 10 mg per mL. The cell concentration was monitored by Trypan blue dye staining using an automated cell counter (Invitrogen™ Countess™, Thermo Fisher Scientific, Waltham, MA, USA).

#### 4.7.2. Antioxidant Activity and MTT Cell Viability Assay

An MTT assay was used to study the S9 and S14 compound’s antioxidant effect after induction of oxidative stress with increasing concentrations of hydrogen peroxide (H_2_O_2_) [66,67]. HT-29 cells were seeded in Falcon^®^ 96-well clear flat-bottom microplates (Becton, Dickinson and Company, Franklin Lakes, NJ, USA) with 200 µL of DMEM medium, and after 24 h of incubation, different concentrations of H_2_O_2_ ranging from 10 µM to 1mM diluted in the medium, together with S9 and S14 HWEs (dil. 1:100), were added to each well in quadruplicate. One quadruplet of each H_2_O_2_ concentration (10 µM, 50 µM, 100 µM, 250 µM, 500 µM, and 1mM) were kept as controls to create an oxidative stress calibration curve. After 24 h of incubation in a humidified atmosphere with 5% CO_2_ at 37 °C, 22 µL of a 5 mg mL^−1^ MTT dye solution was added to each well to reach a final concentration of 0.5 mg mL^−1^. The cells were then incubated in a humidified atmosphere with 5% CO_2_ at 37 °C for 3 h to allow the formation of formazan, which was subsequently dissolved in 150 µL of DMSO at 37 °C for 1 h. After a brief mechanical shaking of the microplates, the optical density at 570 nm was determined using a microplate reader (DV990BV6, GDV, Rome, Italy). All measurements were performed in two independent experiments. A statistical analysis of all comparisons was performed with ANOVA in Microsoft Excel.

#### 4.7.3. Evaluation of Intracellular ROS Production

The H_2_DCFH-DA assay was used to quantify the levels of intracellular ROS. The non-fluorescent probe DCFH-DA easily permeates the cell membrane and is digested by intracellular esterases to produce DCFH. In the presence of ROS, DCFH is subsequently quickly oxidized to form highly fluorescent DCF. HT-29 cells were seeded in Corning 96-well black round bottom polystyrene microplates (Corning Incorporated, Corning, NY) (10 × 10^3^ cells/well) in 200 μL of culture medium (DMEM). The following day, the medium was substituted with a fresh medium containing 100 and 250 µM concentrations of H_2_O_2_ and 1mg/mL of S14. Cells were treated with 10 μM H_2_DCF-DA for 60 min in a humidified environment at 37 °C and 5% CO_2_ after being rinsed with 100 μL of PBS after 24 h. Following that, the wells were washed with 100 μL of PBS and emptied. Finally, 200 μL of PBS were added to each well. At excitation and emission wavelengths of 485 and 530 nm, respectively, the fluorescence intensity of the oxidized form of DCF was quantified using a microplate reader (Beckman Coulter DTX880, Beckman Coulter, Inc., Brea, CA, USA). Data were normalized to cell viability as determined by the MTT assay on additional Falcon 96-well clear flat-bottom microplates (Becton, Dickinson and Company, Franklin Lakes, NJ, USA) seeded with cells that were simultaneously treated and photoexposed under the same experimental conditions. Data were expressed as the percentage of DCF fluorescence intensity with respect to vehicle control.

## 5. Conclusions

Environmentally friendly maceration of hemp residual was tested in this study to obtain valuable new “green” products from low-value starting materials, using only a limited amount of water for the entire process and formulation. Variations in the maceration procedures allowed for the production of five extracts containing bioactive compounds, with antioxidants or anti-biofilm properties. Beyond the specific achievements, this study demonstrates the potential of simple processing systems to obtain good bioactive compounds from waste.

## Figures and Tables

**Table 1:** HWEs are produced after a short- and long-term maceration process in water. S2 and S8 were obtained using mechanical extraction after a short maceration process of 72 h using water as the sole solvent, and pre-treating the waste material with the microwave energy (MWE)-assisted process before the extraction. S9 and S21 were obtained using mechanical extraction after one and two months of water maceration, respectively, without any pre-treatment before the extraction. S14 was obtained with short maceration and protein precipitation in addition to the mechanical extraction method.

HWE ID	Plant Variety	Plant Material	Extraction Method	Other Treatments
S2	*Cannabis sativa* L. cv. Strawberry	Waste dry flowers and leaves	72 h water extraction	Mechanical extraction + MWE-assisted process
S8	*Cannabis sativa* L. cv. Strawberry	Waste green flowers and leaves	72 h water extraction	Mechanical extraction + MWE-assisted process
S14	*Cannabis sativa* L. cv. Strawberry	Waste dry flowers and leaves	72 h water extraction	Mechanical extraction + protein precipitation
S9	*Cannabis sativa* L. cv. Strawberry	Waste dry flowers and leaves	30 days water extraction	Mechanical extraction
S21	*Cannabis sativa* L. cv. Strawberry	Waste dry flowers and leaves	60 days water extraction	Mechanical extraction

**Table 2:** Physicochemical characteristics and biomass analysis of samples S2, S8, S14, S9, and S21.

Sample ID	S2	S8	S14	S9	S21
Conductivity (μS cm^−1^)	205	4870	138.8	9003	10500
pH	8.37	8.11	5.74	6.37	8.16
Fresh weight (g mL^−1^)	0.983	0.983	0.981	1.004	0.967
Dry weight (g)	0.030	0.003	0.011	0.013	0.008
Dry matter (%)	1.0	0.3	1.1	1.30	0.8
Bradford (mg mL^−1^)	0.43	0.72	2.9	0.55	5.7

**Table 3:** *Candida* strains with corresponding collection numbers and identification codes.

Species	Collection n°	ID
*Candida albicans*	CMC2042	CA1
*Candida albicans*	CMC1959	CA2
*Candida tropicalis*	CMC1827	CT1
*Candida tropicalis*	CMC1839	CT2
*Candida tropicalis*	CMC2052	CT3
*Candida parapsilosis*	CMC1973	CP1
*Candida parapsilosis*	CMC2006	CP2
*Candida parapsilosis*	CMC1951	CP3

## Data Availability

The raw data supporting the conclusions of this article will be made available by the authors on request.

## Supplementary Materials

The following supporting information can be downloaded at: https://www.mdpi.com/article/10.3390/ijms25073979/s1.
