# Supplementary material for: Water Extracts from Industrial Hemp Waste Inhibit the Adhesion and Development of Candida Biofilm and Showed Antioxidant Activity on HT-29 Colon Cancer Cells
Source: Int J Mol Sci. 2024 Apr 3;25(7):3979. doi: 10.3390/ijms25073979 (PMC11011686; doi:10.3390/ijms25073979)
Supplement: Supplementary file 1 [file ijms-25-03979-s001.zip › ijms-2924031-SI.pdf]

Supplementary Table S1. The percentage increase or decrease in *Candida* strains growth was observed with each HWE across ten different concentrations compared to the control. Positive values indicate an increase in yeast growth, while negative values signify a decrease. The concentrations of the HWEs ranged from 2.0 mg mL^{-1} to 0.0035 mg mL^{-1}.

HWE	HWE concentration (mg mL^{-1})	CA1	CA2	CT1	CT2	CT3	CP1	CP2	CP3
S2	2	128.71	111.57	1.43	18.34	15.65	63.15	24.72	41.43
	1	125.94	93.58	1.73	27.94	20.92	32.33	20.67	44.39
	0.5	117.68	76.10	-2.61	36.11	10.82	52.51	37.62	49.70
	0.25	101.66	69.06	3.61	42.66	23.54	64.73	31.40	64.75
	0.125	75.18	48.55	1.90	48.81	21.55	58.97	26.75	54.48
	0.065	52.75	37.81	9.23	42.73	21.65	45.55	26.96	43.43
	0.032	31.74	13.71	16.87	28.56	20.45	51.90	17.18	38.90
	0.0155	22.10	8.49	-1.51	29.56	9.48	45.81	-5.74	32.90
	0.007	15.30	6.63	5.41	26.25	10.31	19.44	5.89	29.50
	0.0035	14.23	1.00	1.81	13.10	12.65	31.41	1.95	-8.18
S8	2	189.07	122.96	40.32	31.61	32.73	65.47	92.45	169.41
	1	207.70	112.99	13.19	28.82	33.74	67.51	92.62	170.21
	0.5	151.82	95.55	7.78	44.78	23.99	63.18	90.54	119.45
	0.25	142.42	86.46	21.03	50.38	23.04	61.32	86.54	71.73
	0.125	116.42	87.04	26.70	39.01	3.43	61.32	79.39	52.79
	0.065	126.14	60.15	9.85	36.36	1.77	61.86	62.44	53.70
	0.032	93.57	48.52	23.65	32.62	9.90	48.19	41.39	33.23
	0.0155	84.58	24.37	23.63	20.08	11.69	37.06	20.68	32.73
	0.007	42.51	17.99	12.25	14.23	2.81	19.42	14.80	27.96
	0.0035	47.37	7.05	2.20	11.23	1.73	9.37	5.92	20.11
S14	2	-37.40	-0.25	8.42	49.42	49.52	27.94	0.55	36.04
	1	-28.47	5.10	10.99	15.17	33.66	-5.64	-16.34	26.93
	0.5	-36.99	24.53	11.64	11.28	36.58	-0.33	-14.34	30.37
	0.25	-14.71	26.51	28.02	35.72	14.29	29.47	6.15	25.15
	0.125	-11.96	25.37	34.38	17.12	19.19	16.75	15.74	23.15
	0.065	-17.84	22.42	34.75	15.61	21.44	14.63	19.14	26.37
	0.032	-24.87	10.67	30.38	1.98	-10.87	14.63	-19.34	10.27
	0.0155	-21.41	12.17	29.07	-1.04	-12.58	10.62	-12.84	6.05
	0.007	-5.45	10.72	27.86	-0.60	-7.73	14.04	-9.45	11.49
	0.0035	-19.17	7.38	22.03	-0.60	-0.01	8.27	-19.54	0.17
S9	2	157.50	126.09	58.65	72.12	70.40	82.14	101.05	197.60
	1	155.03	115.14	47.87	61.09	62.66	71.34	95.47	140.31
	0.5	111.10	116.98	24.20	62.62	56.27	76.55	98.51	102.54
	0.25	94.26	96.58	39.74	55.15	48.85	75.30	91.60	50.60
	0.125	79.24	80.98	11.06	48.77	37.62	58.43	88.82	37.64
	0.065	43.58	58.36	20.43	43.08	30.60	52.26	58.45	0.52
	0.032	41.48	23.82	30.28	33.56	41.37	25.70	31.99	5.09
	0.0155	22.40	-3.16	9.53	23.61	37.00	26.07	21.09	6.87
	0.007	20.24	6.90	10.98	13.96	25.08	14.05	10.45	1.21
	0.0035	26.03	18.59	7.69	5.31	27.95	6.77	6.42	3.88

S21	2	94.65	89.98	58.82	110.48	147.26	62.22	174.23	413.65
	1	93.78	77.49	50.89	89.67	136.12	34.19	169.27	282.07
	0.5	99.78	71.63	50.40	78.49	134.41	39.46	122.20	159.95
	0.25	86.63	61.70	43.45	76.64	127.65	26.47	96.28	69.12
	0.125	71.30	54.00	37.04	68.85	115.91	15.33	61.53	30.88
	0.065	46.38	32.13	30.26	53.20	97.36	3.60	56.60	24.01
	0.032	29.13	15.64	13.33	39.01	95.99	15.47	28.57	26.92
	0.0155	20.88	6.02	22.11	34.23	49.57	0.05	23.14	8.63
	0.007	11.48	2.08	15.23	23.20	49.53	0.55	17.80	20.62
	0.0035	3.93	2.01	5.01	17.51	38.84	0.32	6.42	23.54

Supplementary Table S2. The percentage increase or decrease in *Candida* strains biofilm growth was observed with each HWE across ten different concentrations compared to the control. Each HWE was added in the medium of growth for the priming phase of biofilm only. Positive values indicate an increase in yeast growth, while negative values signify a decrease. The concentrations of the HWEs ranged from 2.0 mg mL^{-1} to 0.0035 mg mL^{-1}.

HWE	HWE concentration (mg mL^{-1})	CA1	CA2	CT1	CT2	CT3	CP1	CP2	CP3
S2	2	-4.62	-41.00	23.32	10.60	-22.30	-5.80	5.70	8.70
	1	-8.10	-37.39	28.70	12.66	-23.13	-7.48	1.36	1.83
	0.5	-7.76	-34.51	35.87	7.81	-15.42	-4.42	2.72	3.50
	0.25	-5.24	-30.96	36.63	3.53	-12.79	-3.40	2.92	4.73
	0.125	-7.90	-30.65	29.67	3.52	-8.00	-2.48	5.29	5.62
	0.065	-8.27	-30.92	19.16	-5.04	-8.09	-5.77	3.74	4.23
	0.032	-5.71	-28.95	8.81	-6.51	-5.80	-5.05	1.24	4.56
	0.0155	-4.89	-24.66	4.28	-1.61	-2.26	-2.74	1.68	2.61
	0.007	-3.76	-22.29	2.33	0.17	-6.31	-1.98	0.89	1.17
	0.0035	-3.01	-14.92	-3.89	-0.64	-3.87	-2.94	1.17	2.07
S8	2	1.35	-58.93	0.27	2.50	-35.50	-24.90	-22.90	-42.10
	1	0.04	-57.85	0.30	3.31	-35.93	-26.41	-20.70	-36.24
	0.5	-0.27	-44.35	0.38	-2.72	-31.40	-17.80	-9.49	-23.29
	0.25	-2.87	-36.77	0.27	0.55	-27.60	-12.37	-4.81	-12.67
	0.125	-2.48	-29.44	0.01	-2.87	-16.28	-6.50	-1.49	-6.60
	0.065	-4.17	-26.61	0.16	0.93	-13.72	-4.86	-2.00	-3.76
	0.032	-3.93	-18.18	0.16	-3.16	-10.41	-1.31	-0.95	-1.47
	0.0155	2.36	-14.74	0.23	-0.92	-6.15	1.33	1.18	2.97
	0.007	-1.10	-11.20	0.46	-5.05	-8.60	-0.88	2.30	1.99
	0.0035	0.42	-7.90	0.37	-3.99	-4.11	-0.84	-0.30	2.74
S14	2	10.30	3.38	27.20	6.40	45.20	3.10	6.60	10.40
	1	15.44	7.08	16.87	6.81	36.23	-0.79	6.50	9.63
	0.5	18.44	2.22	16.18	1.75	34.80	-1.29	1.07	8.98
	0.25	17.86	2.32	8.09	3.26	35.56	2.58	3.23	5.77
	0.125	8.61	-9.99	3.58	-2.64	24.56	3.08	3.12	4.63
	0.065	4.32	-6.58	-0.63	-9.01	11.75	-0.71	0.09	0.36
	0.032	-0.39	-9.48	-5.39	-5.01	13.13	-3.66	1.84	1.10
	0.0155	5.22	-7.67	-3.75	-3.69	10.72	0.58	6.55	-0.08

	0.007	-0.93	-7.57	-4.01	1.43	9.22	-1.31	5.26	1.56
	0.0035	-0.80	-3.83	2.56	-0.54	8.63	-1.29	4.77	0.12
S9	2	7.94	-12.59	-38.68	-9.80	-26.30	-21.30	-2.80	60.90
	1	2.13	-2.66	-37.16	-11.68	-19.96	-17.69	0.29	9.78
	0.5	-4.23	-5.20	-26.71	-12.67	-15.39	-13.45	4.74	13.83
	0.25	-3.35	-1.92	-17.68	-11.39	-8.06	-9.29	4.89	13.40
	0.125	-1.31	-1.31	-14.06	-8.21	-7.99	-3.80	8.15	18.50
	0.065	-0.13	5.25	-12.34	-10.92	-7.11	-2.70	2.96	20.78
	0.032	-2.08	-1.39	-11.29	-8.31	-6.90	-0.57	3.47	20.32
	0.0155	0.06	2.64	-8.06	-6.32	-5.34	-0.85	2.90	20.69
	0.007	0.87	2.65	-5.86	-7.44	-6.06	0.88	0.72	23.95
	0.0035	-1.02	0.77	-3.81	-2.06	-2.85	-4.48	1.99	16.79
S21	2	-0.33	-48.98	7.20	23.20	-28.30	-19.80	-11.40	-17.60
	1	-12.92	-43.42	7.69	2.06	-28.52	-12.22	-2.60	1.83
	0.5	-12.66	-43.63	12.17	0.23	-21.15	-4.13	-0.47	8.84
	0.25	-9.60	-37.39	8.20	-1.24	-13.50	0.76	5.32	12.49
	0.125	-6.63	-39.79	6.54	0.17	-8.94	0.97	4.28	12.29
	0.065	-7.75	-33.36	4.45	0.36	0.49	-0.29	2.05	6.85
	0.032	-6.31	-24.99	0.70	-2.14	-1.12	-0.37	1.54	5.20
	0.0155	-0.01	-19.78	2.71	-0.24	5.53	-0.97	2.12	0.51
	0.007	-0.75	-14.93	3.23	3.70	9.57	0.23	3.00	-1.18
	0.0035	0.20	-5.92	3.49	-1.14	-1.67	-2.01	1.86	2.99

Supplementary Table S3. The percentage increase or decrease in *Candida* strains biofilm growth was observed with each HWE across ten different concentrations compared to the control. Each HWE was added in the medium of growth only after the biofilm priming phase. Positive values indicate an increase in yeast growth, while negative values signify a decrease. The concentrations of the HWEs ranged from 2.0 mg mL^{-1} to 0.0035 mg mL^{-1}.

HWE	HWE concentration (mg mL^{-1})	CA1	CA2	CT1	CT2	CT3	CP1	CP2	CP3
S2	2	113.68	-29.22	34.31	74.90	154.30	147.00	95.10	-8.00
	1	45.60	-51.94	8.64	20.61	6.39	29.09	6.39	-2.51
	0.5	3.43	-47.84	12.54	12.96	19.61	34.03	7.91	10.96
	0.25	8.23	-45.96	14.56	15.76	17.59	37.23	8.23	11.65
	0.125	11.69	-40.97	20.84	8.07	17.15	37.39	1.47	11.17
	0.065	3.87	-40.32	5.99	5.37	13.84	43.40	5.07	8.24
	0.032	1.61	-38.72	0.64	-0.53	10.54	39.88	6.39	8.01
	0.0155	5.72	-34.21	-0.47	-2.02	12.82	33.47	4.24	6.88
	0.007	14.59	-21.56	-3.41	-0.29	6.09	24.77	5.35	4.48
	0.0035	4.91	-27.22	-4.55	-4.92	7.51	17.39	4.13	5.23
S8	2	-3.53	-19.37	-25.68	-11.70	-19.40	-32.70	-14.10	-18.50
	1	-5.30	-13.73	-16.81	-17.50	-13.73	-21.99	-13.41	-4.35
	0.5	-5.06	-3.06	-0.88	-15.31	-3.06	-10.35	-6.42	4.67
	0.25	-2.13	3.18	13.49	-14.21	3.18	-4.01	-4.39	6.76
	0.125	-0.32	6.18	19.32	-11.73	6.18	2.27	3.99	8.91
	0.065	-3.38	-0.60	21.92	-10.54	-0.60	8.42	5.07	13.76

	0.032	-0.56	-4.39	14.08	-13.54	-4.39	5.90	5.95	13.56
	0.0155	0.98	-5.86	11.47	-13.89	-5.86	7.02	7.95	14.25
	0.007	-0.81	-8.49	6.67	-20.30	-8.49	-0.55	5.75	9.33
	0.0035	0.85	-5.77	6.01	-18.31	-5.77	-3.56	2.89	6.60
**S14**	2	4.41	-13.16	1.10	43.50	89.20	24.90	6.40	20.30
	1	4.05	-17.98	-3.55	29.25	62.86	22.20	0.87	10.51
	0.5	0.00	-19.86	-4.72	20.57	58.94	22.13	-1.86	8.22
	0.25	2.79	-15.95	-6.22	13.07	33.43	31.13	-1.94	7.80
	0.125	4.55	-12.19	-7.77	13.77	20.87	33.40	-0.75	9.34
	0.065	-5.22	-10.61	-12.01	6.62	8.31	28.30	0.85	10.22
	0.032	-5.23	-0.16	-9.70	4.84	6.71	20.45	0.05	5.91
	0.0155	9.46	10.00	-7.51	11.77	7.01	8.32	0.21	4.09
	0.007	0.71	-22.26	-3.45	7.05	5.74	3.86	-0.69	2.25
	0.0035	1.17	-1.17	2.60	7.77	7.69	0.11	2.40	1.61
**S9**	2	7.36	-60.62	-17.40	-6.30	-22.56	-22.40	-18.50	-10.50
	1	-4.99	-63.86	-11.40	-12.29	-14.02	-11.10	-13.40	-3.06
	0.5	-6.11	-60.32	-2.89	-13.02	-12.53	-0.67	-4.79	5.49
	0.25	-10.61	-52.44	1.47	-9.82	-9.21	12.25	2.80	10.96
	0.125	-11.38	-47.19	-1.82	-10.49	-9.54	13.58	-1.14	11.08
	0.065	-12.62	-42.46	-5.59	-14.03	-9.25	14.90	-4.45	7.64
	0.032	-13.59	-40.25	-7.55	-16.90	-10.93	8.79	-6.03	7.35
	0.0155	-12.98	-34.59	-10.25	-12.11	-8.09	2.89	-4.95	5.66
	0.007	-12.31	-35.79	-9.47	-11.77	-8.24	-3.08	-5.51	8.36
	0.0035	-8.74	-31.14	-6.12	-10.77	-7.29	-3.14	-5.83	-0.32
**S21**	2	-9.18	-56.65	4.60	-7.00	-14.70	-8.70	-20.70	-10.50
	1	-2.08	-59.04	1.69	-13.55	-14.88	5.84	-18.70	-3.06
	0.5	1.14	-56.57	2.11	-12.94	-3.18	13.16	-15.35	5.49
	0.25	2.98	-58.26	6.99	-9.59	-3.23	20.73	-14.12	10.96
	0.125	2.06	-56.00	6.61	-7.88	-1.39	19.54	-12.11	11.08
	0.065	5.27	-55.19	8.84	-7.61	-1.82	10.60	-12.71	7.64
	0.032	1.32	-53.22	8.22	-4.09	-5.70	10.69	-11.87	7.35
	0.0155	1.29	-48.99	8.84	-6.82	-5.95	4.62	-3.22	5.66
	0.007	-1.61	-48.48	1.91	-6.71	-10.52	-0.92	-4.37	8.36
	0.0035	-7.13	-49.83	3.52	4.30	-9.04	0.16	-2.21	-0.32

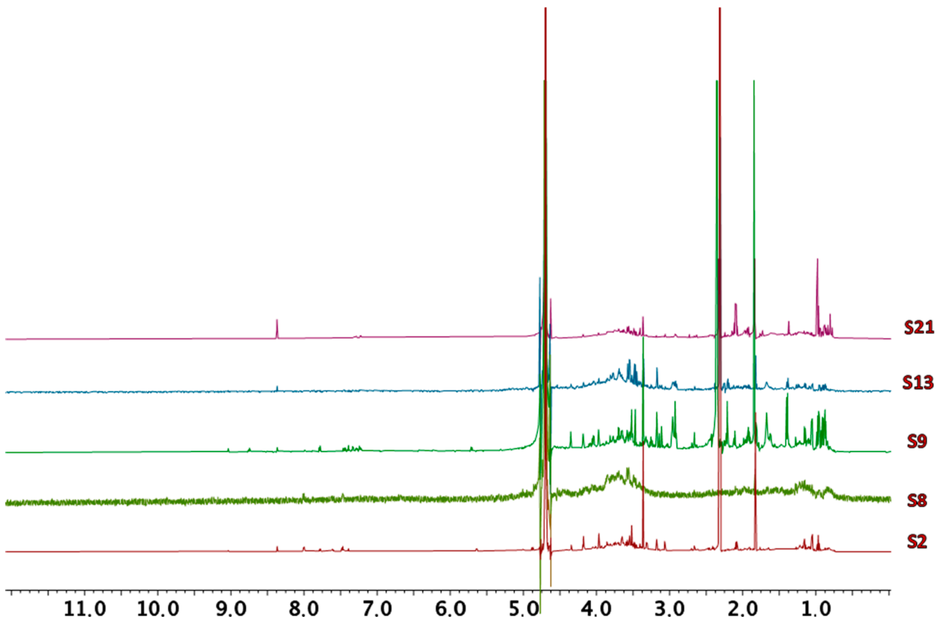

Figure S1. Spectra of samples S2, S8, S9, S14, and S21 acquired in D_2O using ZG pulse program.

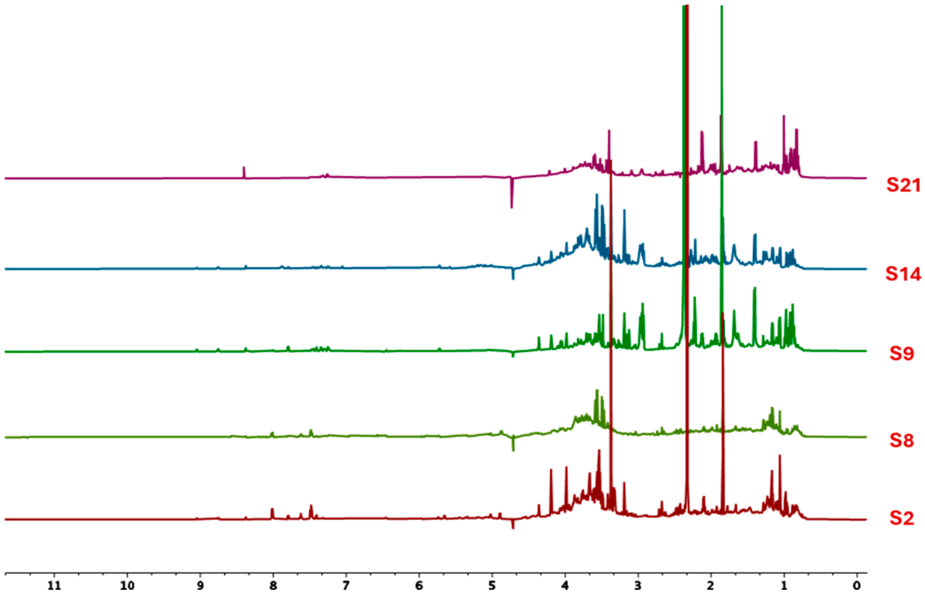

Figure S2. Spectra of samples S2, S8, S9, S14, and S21 acquired in D_2O using AU_WATERSC pulse program.

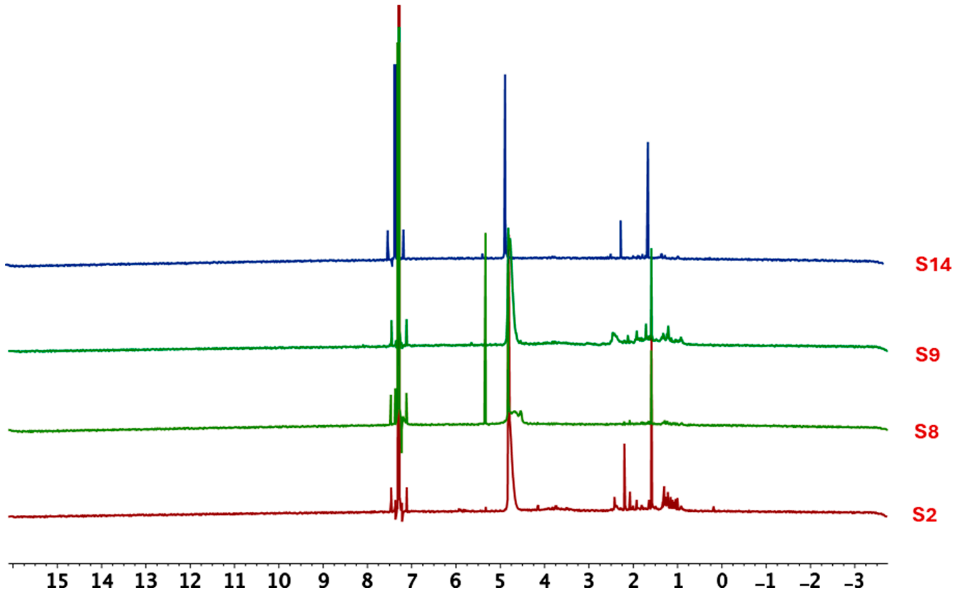

Figure S3. Spectra of samples S2, S8, S9, and S14 acquired in $CDCl_3$ using ZG pulse program.
